# Mesenchymal Stem Cell-Derived Extracellular Vesicles: Hype or Hope for Skeletal Muscle Anti-Frailty

**DOI:** 10.3390/ijms24097833

**Published:** 2023-04-25

**Authors:** Elancheleyen Mahindran, Wan Safwani Wan Kamarul Zaman, Khairul Bariah Ahmad Amin Noordin, Yuen-Fen Tan, Fazlina Nordin

**Affiliations:** 1 Centre for Tissue Engineering and Regenerative Medicine, Faculty of Medicine, Universiti Kebangsaan Malaysia, Jalan Yaacob Latif, Bandar Tun Razak, Cheras, Kuala Lumpur 56000, Malaysia; elan23.97@gmail.com; 2 Department of Biomedical Engineering, Faculty of Engineering, Universiti Malaya, Kuala Lumpur 50603, Malaysia; 3 School of Dental Sciences, Universiti Sains Malaysia, Kampus Kesihatan Kubang Kerian, Kubang Kerian 16150, Malaysia; 4 PPUKM-MAKNA Cancer Center, Universiti Kebangsaan Malaysia Medical Centre, Jalan Yaacob Latif, Bandar Tun Razak, Cheras, Kuala Lumpur 56000, Malaysia; 5 Faculty of Medicine and Health Sciences, Universiti Tunku Abdul Rahman, Sungai Long Campus, Bandar Sungai Long, Kajang 43000, Malaysia

**Keywords:** mesenchymal stem cell, extracellular vesicles, ageing, frailty, skeletal muscle

## Abstract

Steadily rising population ageing is a global demographic trend due to the advancement of new treatments and technologies in the medical field. This trend also indicates an increasing prevalence of age-associated diseases, such as loss of muscle mass (sarcopenia), which tends to afflict the older population. The deterioration in muscle function can cause severe disability and seriously affects a patient’s quality of life. Currently, there is no treatment to prevent and reverse age-related skeletal muscle ageing frailty. Existing interventions mainly slow down and control the signs and symptoms. Mesenchymal stem cell-derived extracellular vesicle (MSC-EV) therapy is a promising approach to attenuate age-related skeletal muscle ageing frailty. However, more studies, especially large-scale randomised clinical trials need to be done in order to determine the adequacy of MSC-EV therapy in treating age-related skeletal muscle ageing frailty. This review compiles the present knowledge of the causes and changes regarding skeletal muscle ageing frailty and the potential of MSC-EV transplantation as a regenerative therapy for age-related skeletal muscle ageing frailty and its clinical trials.

## 1. Introduction

The global demographic pattern involves a growing number and proportion of elderly persons in the population. The proportion of people 65 years of age and older worldwide is predicted by the United Nations (UN) to double by 2050, reaching almost 1.5 billion elderly people globally [1]. Age-related frailty is a major public health concern globally as the global geriatric population rises, especially in nations with the longest life expectancies [2]. Frailty is defined as an age-related decline in the functional reserve of multiple body systems that results in a reduced ability to cope with acute or external stressors [3]. Frailty is indicated by easy exhaustion, diminished libido, emotional disruption, accelerated osteoporosis, impaired muscle strength, and vulnerability to illness [4]. A person is more likely to progress up the Clinical Frailty Scale as they age, which correlates to illnesses that have higher morbidity and mortality rates [5]. Several age-related conditions involving oxidative stress, such as cardiovascular diseases [CVDs], chronic obstructive pulmonary disease (COPD), chronic kidney disease (CKD), neurodegenerative diseases, and cancer, including sarcopenia and frailty, are more common in the elderly [6]. The prevalence of these chronic degenerative diseases will rise over time, placing a significant strain on the global healthcare industry to manage the diseases.

Meanwhile, skeletal muscle ageing frailty, which is defined as a loss in muscular mass, strength, and function, is a prevalent condition among older persons [7]. As a substantial clinical syndrome linked to an elevated incidence of falls, depression, and disability, which increases mortality, skeletal muscle ageing fragility is of increasing importance [8]. As a result of ageing, muscle mass declines naturally, beginning in the late twenties and accelerating in the fifties [9]. Sarcopenia, an age-related gradual loss of muscle, is one way that muscle loss can appear.

Many of the health issues that arise as people age are linked to chronic illnesses, especially degenerative illnesses, and can be avoided or delayed by adopting healthy behaviours. Indeed, both physical activity and a healthy diet have a significant positive impact on one’s health and wellbeing [10]. Pharmacological therapies can be used to effectively control other health issues and capacity deficits, especially if they are caught early [3]. There is, however, still no known treatment for this illness. The use of stem cells to treat a variety of illnesses and disorders has recently shown promising outcomes. Since frailty is also linked to stem cell depletion and exhaustion, where the stem cells’ activity is characterised by decreased survival, proliferation, differentiation, and homing capacity [11,12], cell-based therapy represents a viable strategy to be able to treat or prevent the development of frailty [13].

Mesenchymal stem cells (MSCs) have recently emerged as promising candidates for treating a variety of age-related conditions, including ageing frailty. MSCs can differentiate into different cell lineages and secrete extracellular vesicles (EVs), such as exosomes and microvesicles, that contain bioactive molecules, such as proteins, nucleic acids, and lipids. These EVs can deliver cargo to target cells and influence cellular processes, such as inflammation, apoptosis, and angiogenesis, promoting tissue repair and regeneration [14].

Ageing and pathophysiological changes associated with ageing are unavoidable. There is no effective therapy for age-related pathophysiological changes, such as sarcopenia, other than physical activity and good nutrition. MSC-EV therapy has shown great promise in slowing the progression of these age-related pathophysiological changes. The potential use of MSC-EVs to rejuvenate ageing muscle fibre cells and increase the bioenergy level of ageing skeletal muscle is the focus of this paper.

## 2. Mesenchymal Stem Cell-Derived Extracellular Vesicles (MSC-EVs)

**Figure 1:**
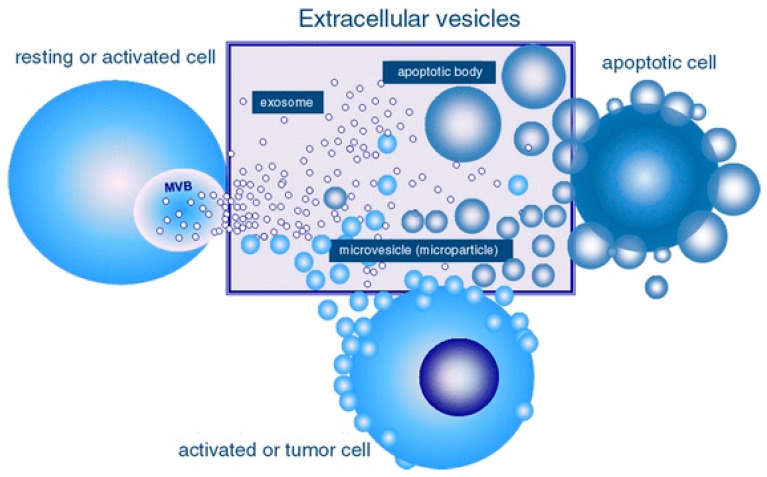
Schematic representation of the extracellular vesicles. Major populations include exosomes, microvesicles, and apoptotic bodies. Source: György, Szabó, Pásztói, Pál, Misják, Aradi, László, Pállinger, Pap, Kittel, Nagy, Falus and Buzás [17].

In recent years, researchers have focused on the indirect use of MSCs, which is based on extracellular vesicles (EVs) derived from these cells [15]. Apoptotic bodies, microvesicles (MVs), and exosomes are three types of EVs that differ in size, content, and formation [16], as illustrated in Figure 1 [17]. Apoptotic bodies are 50–4000 nm in size and are typically produced by apoptotic cells in the final stage of apoptosis. These EVs are diverse, containing membrane components (such as phosphatidylserine), nuclear material, and cellular organelles [18]. Microvesicles, unlike apoptotic bodies, shed directly from the membrane of healthy cells. These EVs, like the apoptotic body, have a heterogeneous morphology and range in size from 100 to 1000 nm. Microvesicles can influence gene expression by sending miRNA to neighbouring cells. Furthermore, because MVs are not released from the cell via endocytosis, they lack endocytosis-related proteins [19]. Exosomes are the smallest EVs, measuring 30–120 nm in size, and are formed during late endosome membrane inward invagination and the formation of multiple vesicular bodies (MVBs) [20]. Exosomes are formed inside MVBs and secreted to the extracellular environment via endocytosis by the MVB membrane fusing with the cell membrane [21]. Exosomes are now classified into three types based on their size: large exosomes (exo-L, size is between 90 and 120 nm), small exosomes (exo-S, size is between 60 and 80 nm), and exomers (35 nm) [22].

### 2.1. Isolation of MSC-EVs

MSCs produce more exosomes than other cells, making them clinically viable for exosome separation and therapy [23]. For example, tetraspanins (CD63, CD9, CD81, CD82), fusion-involved proteins (flotillins, CD9, annexin, GTPases), adhesion molecules, gap junction related proteins (Connexins-43) [24], heat shock proteins (HSC70 and HSC90), MHC-1, MHC-2, membrane transporters (GTPases), Rab proteins [25], lysosomal proteins (Lamp2b), and proteins involved in multivesicular body biogenesis (Alix and TSG101) [26,27].

Exosomes are separated using various methods, including ultracentrifugation, density gradient centrifugation, pegylation-based methods, and kit use. There are several relatively efficient protocols available, such as 100,000× *g* ultracentrifugation of complete medium (or serum after at least 1:4 dilution) for at least 18 h [28], centrifugation at higher speeds (e.g., 200,000× *g* [29]) for shorter periods of time, or tangential flow filtration or other forms of ultrafiltration [30]. A few hours of ultracentrifugation at around 100,000× *g* without dilution will not eliminate all EVs or EV-associated RNA [31,32,33].

### 2.2. Characterisation of MSC-EVs

Exosomes can be used for therapeutic purposes after being characterised using various methods, such as dynamic light scattering (DLS), scanning electron microscopy (SEM), transmission electron microscopy (TEM), and ELISA. Exosomes do not pose a risk of genetic instability or immunosuppression after allogeneic administration in in vivo models. Exosome therapy has been shown in studies to be a new strategy for overcoming stem cell therapy deficiencies [34].

### 2.3. Therapeutic Effects of MSC-EVs

Exosomes are cellular communication vesicles that are paracrine. A lipid bilayer membrane can transport cytokines, chemokines, growth factors, various enzymes, various signalling molecules, miRNAs, lipids, and transcription factors. According to research, cargos present in MSC exosomes include ATP synthesis enzymes (glyceraldehyde 3-phosphate dehydrogenase (GAPDH)), phosphoglycerate kinase (PGK), phosphoglucomutase (PGM), enolase (ENO) [35], angiogenesis stimulating enzymes (VEGF, inducer extracellular matrix metalloproteinase (EMMPRIN), and MMP-9) [36], various transcription factors (transcription factor with Octamer 4 (Oct-4), HoxB4, and Rex-1) [34], tumour growth inhibitory miRs (miR-23b, miR-214, miR-451, miR-223, MiR-31, miR-24, miR-125b, and miR-122) [37], and inflammation regulating miRs (miR-155 and miR-146) [38]. Hence, bilayer lipids protect nucleic acids and proteins from extracellular degradation, allowing for efficient transport.

Exosomes are smaller and less complex than their parent cells, and their membranes contain less protein. As a result, they are easier to separate and store, and they are less immunogenic than cell therapy [39]. Exosomes are also less likely to become trapped in the lungs or liver. Exosomes can communicate information in a variety of ways, including juxtacrine and solution signalling [40]. Exosomes have several advantages over their source cells: (1) Their use prevents the transfer of cells containing immunogenic molecules as well as mutated or damaged DNA; (2) Exosomes are nano-sized and can easily enter and move within any organ, whereas cells are larger and cannot migrate to the site of injury through capillaries; (3) Exosomes can migrate to different parts of the body due to the presence of homing molecules on their surface; and (4) Because exosomes are native to the body, their surface has biochemical properties similar to those of their derived cells, allowing them to avoid phagocytosis, cell membrane fusion, and lysosomal fusion [41].

Because of the mentioned characteristics, MSC-derived exosomes have emerged as one of the most dynamic fields in regenerative medicine. One of the most common causes of function loss in many chronic degenerative diseases is tissue destruction. The function of these tissues can be rejuvenated if treated with a therapeutic agent. MSC-derived exosomes have been shown to be therapeutic in heart, kidney, lung, skin, brain, liver, autoimmune, and musculoskeletal diseases [42]. Type 1 diabetes, macular degeneration, chronic kidney disease, ischemic stroke [43], Alzheimer’s [44], multiple sclerosis [45], sepsis, hepatitis [46], chronic liver disease [39], and skin disease [47] can all be treated with these exosomes.

Exosomes derived from MSCs have been shown to modulate the immune system, stimulate cell proliferation, promote angiogenesis, prevent apoptosis, and suppress oxidative stress [48]. These exosomes aid in the maintenance of homeostasis and cell repair by providing and transporting active enzymes that restore normal cell activity [49]. Proteomic studies of MSC exosomes revealed the presence of over 200 immunomodulatory molecules [50]. These exosomes also promote cell proliferation and prevent apoptosis by activating the Ras/Raf/MEK/ERK and PTEN/PI3K/AKT/mTOR signalling pathways [51]. Aside from their therapeutic potential, MSC-derived exosomes can migrate to lesion sites. Exosome surface molecules can also be modified to migrate more and better to the site of injury [52]. This feature of exosomes makes them an excellent vehicle and transport system for delivering drugs directly to the site of the disease [53]. Intravenous, intraperitoneal, or subcutaneous exosome injections result in the rapid clearance of exosomes from the bloodstream and accumulation in the liver, spleen, lungs, and gastrointestinal tract [54,55]. Furthermore, regardless of the injection route, the majority of systemically injected exosomes are quickly taken up by macrophages in the reticuloendothelial system and eliminated from the body [56]. As a result, the biological distribution of exosomes following systemic administration can be classified into two stages: (1) Rapid distribution in the liver, spleen, and lungs 30 min after administration, and (2) Exosome removal via hepatic and renal processing 1 to 6 h after administration [57]. Exosomes administered topically (such as the skin surface and ocular surface) have a shorter half-life due to fluid cleansing (sweat and tears) and exposure to external factors [58].

## 3. MSC-Derived EV Therapy for Skeletal Muscle Ageing Frailty

MSC-EVs have been demonstrated in studies to improve skeletal muscle frailty and have been transplanted into frail individuals. MSCs are drawn to injury sites, where they reduce inflammation and promote cellular repair [59]. Remarkably, MSCs demonstrated improved outcomes in frail patients by lowering TNF- and CRP levels and were safe in all patients [59,60]. Before delving into the mechanisms of action of MSC-EV therapy, it is necessary to first understand the age-related pathophysiological changes that occur in skeletal muscle.

### 3.1. Skeletal Muscle Ageing Frailty

**Figure 2:**
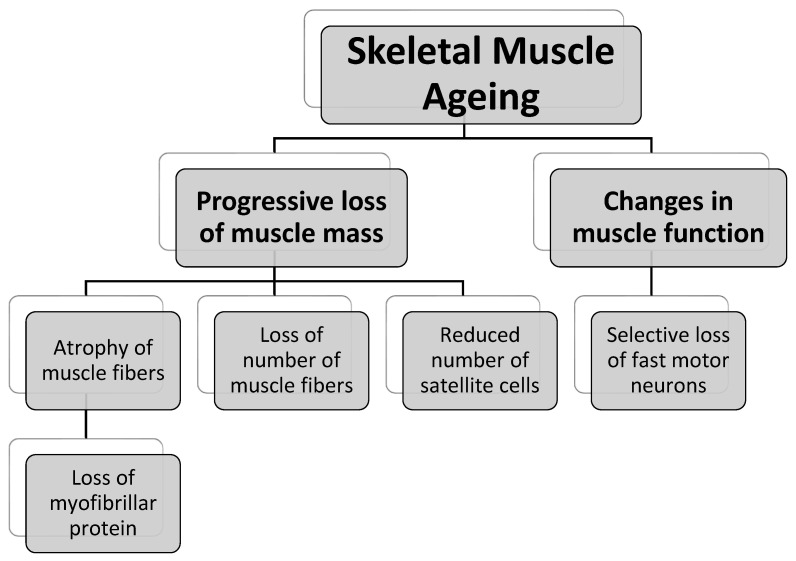
Summary of ageing-related pathophysiological changes in muscle.

Muscle ageing is associated with a gradual decline in skeletal muscle mass and function. Myofibers, which are multinucleated syncytial cells with contractile proteins in their cytoplasm, make up the skeletal muscle. Myofibers are classed as slow twitch (type I) or fast twitch (type II) depending on whether they use aerobic (type I) or anaerobic (type II) metabolism. Ageing causes significant functional muscle strength reduction. Muscle strength can be measured in a variety of ways, including the maximum weight moved in a resistance exercise, the maximum torque produced eccentrically, isometrically, or concentrically, the maximum power produced, or the rate of force development (RFD), all of which have a negative relationship with age [61,62]. In particular, the muscle’s ability to create ‘rapid strength’ (power or RFD) is considerably impaired [63], whereas ‘slow strength’ is less severely weakened. Muscle endurance, or fatigue resistance, on the other hand, is not decreased to the same extent as muscle strength [9]. Overall, these functional alterations in the muscle can be explained by a few biochemical changes, as shown in Figure 2.

#### 3.1.1. Progressive Loss of Muscle Mass

A loss of muscle mass with ageing can be linked to atrophy and a loss of muscular fibres. In general, muscle fibre atrophy occurs as a result of myofibrillar protein loss caused by decreased synthesis of myofibrillar and mitochondrial proteins with age [64]. This is most noticeable in fast (type II) fibres, which show 15–25% atrophy that is more pronounced in the extremely fast type IIX fibres than in the type IIA fibres, but slow (type I) fibres show no substantial loss [65,66]. The decrease in myofibrillar and mitochondrial protein synthesis is due in part to ageing-related endocrine alterations, particularly the decreased production of anabolic cytokines, such as insulin-like growth factor 1 (IGF-1), in ageing muscle [67]. Aged muscles also exhibit ‘anabolic resistance’, which means they become less receptive to anabolic stimuli, such as exercise or amino acid ingestion, both of which enhance protein synthesis [68]. In addition to individual fibre atrophy, there is a general decline in the number of muscle fibres [69]. Essentially, these biological alterations at the muscle fibre level can be explained in part by the death of motor neurons (denervation) and a lack of reinnervation, which leads to muscle fibre atrophy or apoptosis [70].

Another cause of muscle mass loss is ageing impairments in satellite cell activity. Satellite cells are in charge of skeletal muscle regeneration, where they repair injured muscle and help to maintain muscular mass [71]. As ageing occurs, the number of satellite cells decreases by up to 50%, resulting in a loss in muscle regeneration potential [72]. In vitro, aged satellite cells showed decreased activation, proliferation, colony formation, and differentiation [73]. Jejurikar et al. [74] showed that aged satellite cells are more prone to senescence and apoptosis. Furthermore, Chakkalakal et al. [75] demonstrated that with ageing, the satellite cell niche produces higher levels of fibroblast growth factor 2 (FGF2), which leads to a loss of quiescence and self-renewal ability of satellite cells, making them more vulnerable to environmental stresses, such as oxidative stress.

#### 3.1.2. Changes in Muscle Function

Muscle strength and function loss occur at a higher rate than muscle mass loss and have a substantial impact on the elderly. There are several factors underlying muscle function loss, but the most important aspect is the selective loss of fast muscle fibres as a result of the selective loss of fast motor neurons with ageing, which causes fast muscle fibres to be ’orphaned’, and they are then mostly re-innervated by neurons from neighbouring slow motor units, causing them to regroup and partially convert to slow fibres, resulting in a hybrid fibre phenotype or fibre-type switch [69,70]. As a consequence, normal motor unit recruitment is disrupted, and the typical intermixed pattern of muscle fibre types is lost, resulting in a decrease in motor skills [76]. Furthermore, the increase in fibrofatty tissue within skeletal muscle with age causes the disarrangement and modification of the muscular architecture, as well as loss of muscle function. Furthermore, with ageing, there are intrinsic changes in muscle fibres, such as mitochondrial function defects and increased generation of reactive oxygen species [77] as well as changes in the function and relative amounts of mitochondrial proteins [64], resulting in lower respiratory capacity, decreased ATP levels, decreased fatty acid metabolism, intracellular lipid accumulation, and eventual insulin resistance [78].

### 3.2. Protective Effects of MSC-Derived EVs on Ageing Muscle

MSC-derived extracellular vesicles (EVs), which include exosomes and microvesicles (MV), play an important role in intercellular communication, cell signalling, and modifying cell or tissue metabolism over short or long distances in the body. MSC-derived exosomes contain cytokines and growth factors, signalling lipids, mRNAs, and regulatory miRNAs [79,80]. The applications of MSC-EVs in skeletal muscle in both in vitro and in vivo studies are summarised in Table 1.

According to Nakamura et al. (2015) [81], purified MSC-derived exosomes increase skeletal muscle regeneration by enhancing myogenesis and angiogenesis, which is mediated by miRNAs, such as miR-494, in a mouse model of cardiotoxin-induced muscle injury. EVs can promote regeneration in muscle damage models, such as ischemia [82], torn rotator cuffs [83,84], or muscle laceration [85], by increasing angiogenesis and satellite cell activation [85] and decreasing inflammation and fibrosis [81].

In a mouse model of cardiotoxin-induced muscle injury, Lo Sicco et al. (2017) [86] evaluated the anti-inflammatory activities of EVs extracted from adipose tissue-derived MSCs (ASCs) cultivated under normoxic or hypoxic conditions in a mouse model of cardiotoxin-induced muscle damage. The scientists discovered that EVs derived from hypoxia MSCs have stronger anti-inflammatory effects than EVs derived from normoxic MSCs. EVs derived from ASCs cultured under normal conditions were more efficient than EVs isolated from ASCs cultured under hypoxic settings in enhancing survival and decreasing the inflammatory response in rats after a generated sepsis syndrome [87]. In summary, preconditioning MSCs to create more therapeutically effective EVs may be relevant, but further study is needed to clarify what sort of MSCs pre-treatment is required and whether it should be tailored to the type of injury targeted by the EV-based therapy.

In a study conducted by Li et al. (2021) [88], bone marrow mesenchymal stem cell (BMSC)-derived exosomes (Exos) were demonstrated to decrease muscle atrophy, both in vitro and in vivo. C2C12 (subclone from a myoblast cell line established from normal adult C3H mouse leg muscle) myotubes demonstrated a decrease in diameter when treated with dexamethasone (DEXA), which was decreased when C2C12 myotubes were co-cultured with BMSC-Exos. The underlying mechanisms of the BMSC-Exos intervention included the upregulation of miR-486-5p (a microRNA that enhances muscular function and strength when overexpressed) and downregulation of FoxO1 (a transcription factor that plays a role in muscle atrophy). BMSC-Exos prevented DEXA-induced muscle atrophy in mice. A miR-485-5p inhibitor, on the other hand, was demonstrated to reverse such inhibition both in vitro and in vivo. The study concluded that BMSC-Exos inhibited DEXA-induced muscle atrophy via the miR486-5p/FoxO1 axis.

Sahu et al. (2021) [89] demonstrated that circulating EVs in young mice plasma could renew aged muscle cells and improve the muscle regenerative capacity of elderly mice, resulting in an increase in fibre size, muscular force, and mitochondriogenesis with a decrease in fibrosis. They argued that Klotho transcripts (which drop with age) found in juvenile EVs were the main cause of the observed effects. More recently, we found that EVs derived from young ASCs improved physical performance and reduced frailty in old mice. These effects were accompanied by an increase in fibre size and muscle protein content, as well as a decrease in muscle senescence and SASP factors, oxidative stress, and lipid deposition [90]. However, the mechanism of action remains unclear, as senolytic activity could not be found. They may probably act as senomorphics, that is, molecules that suppress the senescent phenotype without the specific induction of apoptosis in senescent cells, probably through the inhibition of the SASP, as has been suggested by Dorronsoro et al. [91].

**Table 1:** Summary of applications of MSC-EVs in skeletal muscle.

Type of MSC-EVs	Key Findings	Mechanisms	References
**In Vitro studies**			
BMSC-Exos	- Promotion of proliferation and differentiation of C2C12 cells - Significant increase in myogenesis and angiogenesis in migration and tube formation assay using HUVECs	Upregulation of miRNAs, such as miR-494	Nakamura et al. (2015) [81]
ASC-EVs	Enhanced myoblast proliferation and differentiation in C2C12 cells ischemia/reoxygenation model	Upregulation of CdK6, Cyclin D1 and p-p38 for proliferation as well as MyoD, Myf5, and Pax7 for differentiation	Figliolini et al. (2020) [82]
ASC-EVs	Efficient internalisation by responding cells, inducing increase in their proliferation rate, and shifting the balance toward an M2 pro-resolving phenotype when co-cultured with bone marrow-derived macrophages	MiRNAs involved in different stages of the healing process	Lo Sicco et al. (2017) [86]
BMSC-Exos	Inhibition of reduction in C2C12 myotubes diameter induced by dexamethasone	- Upregulation of miR-486-5p - Downregulation of FoxO1	Li et al. (2021) [88]
**In Vivo studies**			
BMSC-Exos	Accelerated histological muscular regeneration, with enhanced angiogenesis and reduced fibrosis in mouse muscle injury model	Upregulation of miRNAs, such as miR-494	Nakamura et al. (2015) [81]
ASC-EVs	Reduction in muscle function impairment and protection against muscle damage by acting both on tissue microvessels and muscle cells in mouse hind limb ischemia model	Increased levels of neuregulin 1 (NRG1)	Figliolini et al. (2020) [82]
ASC-Exos	Prevention of muscle atrophy, fatty infiltration, inflammation, and vascularization as well as increased myofiber regeneration and biomechanical properties in rat massive rotator cuff tear (MRCT) model	Suggestive of anti-inflammatory, anti-apoptotic, and proregenerative effects of ASC-Exos	Wang et al. (2019) [84]
ASC-Exos	Prevention of fatty infiltration, promotion of tendon-bone healing, and improvement of biomechanical properties in rabbit chronic rotator cuff tears (RCT) model	Promotion of the formation of fibrocartilage at the tendon-bone interface	Wang et al. (2020) [83]
ASC-EVs	Downregulation of interleukin 6 (IL6), indicating decreased inflammation in mouse muscle injury model	- Activation Nos2 and upregulation of Arg1 and Ym1 - Accelerated expression of the myogenic markers Pax7, MyoD, and eMyhc	Lo Sicco et al. (2017) [86]
BMSC-Exos	Inhibition of dexamethasone-induced skeletal muscle atrophy in mice	- Upregulation of miR-486-5p - Downregulation of FoxO1	Li et al. (2021) [88]
Young ASC-EVs	Improvement in motor coordination, grip strength, fatigue resistance, fur regeneration, and renal function in aged mice	Proregenerative effects and decrease in oxidative stress, inflammation, and senescence markers in muscle and kidney	Sanz-Ros et al. (2022) [90]

Evidence suggests that EVs have several advantages over MSC transplantation. After transplantation, cell degeneration or senescence in the host is not a problem for EV treatment. It has also been found that EVs have fewer cell surface proteins than stem cells, making allogeneic EVs safer than allogeneic stem cells due to the lower risk of immunogenicity [92]. The irreplaceability of EVs and their lack of DNA significantly lowers the likelihood of DNA mutation and tumour formation in the host. When compared to bigger stem cells, the use of EVs reduces the potential for vascular constriction. The bioactive components of EVs can be easily regulated by cultured cells in various situations. EVs are also easier to store than stem cells, as stem cells must be maintained in liquid nitrogen to retain viability, whereas EVs can be stored at −20 °C. Finally, unlike stem cells, the requirement to examine the safety and dose of EVs is less stringent, making the path to the clinical setting smoother and faster. Because stem cells are living cells, predicting the fate of transplanted cells is more challenging.

### 3.3. Current Challenges in Clinical Applications

Despite the therapeutic success of MSC-EVs in preclinical studies, the use of these EVs in clinical settings will require the resolution of several critical issues, such as (i) large-scale production and isolation methods, (ii) methods for rapid and accurate quantification and characterization of EVs, (iii) precise content characterization of the cargo, (iv) pharmacokinetics, targeting, and transfer mechanisms of EVs to the target sites, and (v) safety profiles to determine the optimal clinical dosage and possible toxicities upon repeated administration.

#### 3.3.1. Large-Scale Production of MSC-EVs

Conventional cell maintenance and expansion methods use a two-dimensional culture methodology. Long-term passaging to produce large amounts of EVs may lead cells to lose their clonal and differentiation capacity [93]. As a result, there is an urgent need to discover procedures for dependable MSC expansion in order to bulk-manufacture EVs for clinical use. The existing methods of MSC growth are time-consuming and entail numerous processes. Traditional tissue culture techniques in flasks [94,95] or three-dimensional culturing bioreactors made of polysulphone hollow fibres with semi-permeable membranes that greatly increase the surface area, as described by Mennan et al. (2019) [96] as well as McKee and Chaudhry (2017) [93], are available for MSC culture expansion. Unfortunately, conventional EV production methods have low yields and are not scalable, hindering the advancement of preclinical and clinical usage of EVs as medicines [97]. Massive or multi-layer culture flasks, fixed-bed bioreactors, in-stirred tank bioreactors, or continuous production in perfusion reactors are used in large-scale EV production [98]. When compared to standard planar cell culture in flasks, the majority of these approaches strive to maximise EV generation by maximising the culture surface area [99]. A recent study found that cultivating hUCMSCs in scalable microcarrier-based three-dimensional cultures resulted in a twenty-fold higher EV output than in two-dimensional cultures [100].

Many technical aspects must be standardised when utilising cell culture supernatants for EV extraction to assure batch-to-batch reproducibility and lot-consistent EV generation [101]. Several parameters, including cellular confluence, early vs. late cell passage, oxygen concentration, cytokines, heparin, and serum content of the medium, might influence the quality and quantity of EVs generated by MSCs [102]. For example, studies reveal that foetal bovine serum (FBS), a feed used to grow cells in culture, contains RNA-containing EVs that can impact cell culture behaviour, emphasising the need to devise a technique for producing EVs free of such interferences [31]. Serum-free cultures have also been demonstrated to affect the EV amount and protein composition [103]. Pachler et al. (2017) [104] addressed this issue by developing a Good Manufacturing Practice (GMP)-grade standard protocol in which they demonstrated that hBMMSCs cultured in EV-depleted medium with reduced pooled human platelet lysate (a serum-free medium) (i) retained their morphology, phenotype, viability, and differential potential, (ii) strongly affected hBMMSC proliferation and differentiation capacities, and (iii) were enriched [104]. This paper proposes a method for the large-scale, GMP-compliant manufacture of MSCs and MSC-EVs. Aside from changing the culture conditions, changing the biology of the EV-biogenesis may boost the EV yield [105].

#### 3.3.2. Effective and Scalable EV Isolation Methods from MSCs Culture Medium

Scalable EV isolation procedures, in addition to large-scale EV manufacturing, are absent, making the clinical translation of EV treatments difficult. There are now several methods for isolating EVs [106,107]; however, there is no cutting-edge technology for isolating EVs in large amounts for therapeutic usage. There are five main isolation methods used in research: (i) differential centrifugation, (ii) density gradient ultracentrifugation, (iii) size-exclusion chromatography (SEC), (iv) precipitation, and (v) immune-based capture approach [98]. Interestingly, multiple studies [108,109,110] have shown that isolating EVs from stem cell cultures through ultrafiltration followed by SEC leads to a better yield while keeping the biophysical and functional features of the EVs [111].

Stranska et al. (2018) [112] investigated the popularity of SEC in both therapies and biomarker development for illness diagnostics to demonstrate the superiority of SEC qEV (Izon Science, Lyon, France) over the affinity-based EV separation approach (using exoEasy kit, Qiagen, Hilden, Germany) from human plasma. Surprisingly, SEC alone is incapable of distinguishing plasma EVs from lipoproteins until it is paired with density gradient isolation [113].

#### 3.3.3. Biodistribution and Targeting of MSC-EVs to Target Tissues

It is crucial to address MSC-EV biodistribution and targeting mechanisms in vivo while investigating them as a therapeutic approach. Optical imaging (OI) is one way to explore various tissue targets in living animals. This non-invasive approach can be used to observe tagged cells in vivo using near-infrared (NIR) dyes that optimise the depth of tissue penetration and reduce the background [114,115,116,117,118]. Grange et al. (2014) [115] labelled MSC-EVs in a mouse model of acute kidney injury (AKI) using two methods: the direct labelling of pure EVs and the production of labelled EVs from MSCs pre-incubated with NIR dye. They discovered that EVs were identifiable in whole-body pictures and dissected kidneys using OI and that EVs that were directly tagged with NIR dye showed stronger and brighter fluorescence than MSC-labelled EVs. In addition, they discovered that MSC-EVs accumulated in the kidneys of AKI mice but not in the controls. MSCs are recruited to areas of injury by receptor-mediated interactions [119]. Hence, MSC-EVs, which have the same membrane receptors as MSCs, may be recruited via the same process [115].

Researchers utilised various colours to track the biodistribution of EVs following delivery. Wen et al. (2019) [118] examined the distribution of DiD (1,1′-Dioctadecyl-3,3,3′,3′- Tetramethylindodicarbocyanine, 4-Chlorobenzenesulfonate) lipid dye-labelled MSC-EVs in mice under various settings. The DiD-labelled MSC-EVs were found to be most abundant in the liver and spleen, least abundant in the bone marrow of the spine, femur, and tibia, and undetectable in the lung, heart, and kidney [118]. MSC-EVs are usually labelled with PKH-26A, a lipophilic dye that integrates into cell membranes [120,121,122]. Bucan et al. (2019) [120] investigated the effects of MSC-EVs generated from rat adipose-derived MSC-EVs (rAMSC-EVs) on sciatic nerve regeneration and neurite development. rAMSC-EVs improved sciatic nerve regeneration in vivo following damage and neurite development in vitro. In addition, they identified brain growth factor transcripts in rAMSC-EVs [120]. Wang et al. (2019) [123] also employed DiO (3,3’-Dilinoleyloxacarbocyanine Perchlorate) to identify MSC-EVs in a rat carotid artery balloon injury model. They discovered that MSC-EVs can transmit miR125b to vascular smooth muscle cells, which can slow neointimal development and may be a therapeutic target for vascular disorders [123]. There have also been reports of labelling MSC-EVs with other labelling agents, such as DiI (1,1′-Dioctadecyl-3,3,3′,3′-Tetramethylindocarbocyanine Perchlorate), Alexa fluor 488, and gadolinium in order to locate the biodistribution of EVs [124,125,126].

In addition, Moon et al. (2019) [127] studied the biodistribution, therapeutic effectiveness, and mode of action of MSC-EVs in a preclinical rat stroke model. To label EVs for in vivo tracking, this work employed PKH26 or 5-(and-6)-carboxyfluorescein diacetate succinimidyl ester (CFSE). EVs were detected and counted using flow cytometry, and the size and shape were measured using NanoSight nanoparticle tracking analysis [127]. The MSC-EVs were discovered to have moved to the infarcted brain. Although MSC-EVs accumulated in the infarcted brain in a dose-dependent manner, injected MSCs accumulated in the lung and liver with increasing doses, underlining the fact that MSCs seldom reach target organs [128].

The mechanism of the therapeutic action of EVs is currently unknown. Membrane proteins, cytoplasmic proteins, mRNAs, and microRNAs can all be transferred to target cells via EV cargo. The therapeutic action of EVs is thought to be due to the transfer of miRNAs to diseased and wounded cells [17]. According to research, miRNAs in MSC-EVs regulate the physiology and pathology of microenvironments [127,129]. Furthermore, MSC-EV miRNAs have been demonstrated to regulate heart regeneration and protection [130]. There are methods for loading and altering the EV payload, including electroporation, freeze-thaw cycles, saponin-mediated loading, and hypotonic dialysis [131,132]. EV cargo may also have an impact on EV migration. MSC-EVs are thought to have chemokine receptors that allow them to be targeted to wounded areas [127,133]. Phosphatidylserine-binding and HER2-targeting proteins on the EV surface have been found to improve EV transport to HER2-expressing cells [134]. Previous research has shown that this way of directing EVs to certain tissues is feasible [135,136,137];0 therefore, these strategies might be applied to MSC-EVs as well. Whilst the precise process is unknown, MSC-EVs are thought to function similarly to MSCs. MSCs have therapeutic benefits by secreting substances that minimise cellular damage and promote repair, and MSC-EVs may serve as communication vehicles for MSCs to signal support from the tissue microenvironment [138,139].

#### 3.3.4. Safety Profile

A safety profile must be defined for any therapeutic treatment. While EV-based treatment is still in its early stages, we know that many of the negative consequences of cell therapies are not present in EV-based treatments. The biggest concern about employing stem cell treatment is the potential for transplanted MSCs to suppress anti-tumour immune responses and act as a progenitor for blood vessels, which could encourage tumour development and spread [34]. MSCs are also hampered by tumorigenicity, immunogenicity, and genomic mutability [140,141,142]. Luckily, the constraints listed above do not apply to MSC-EVs. EVs (not created from MSCs) have been used in a few clinical trials, and these studies have demonstrated good safety profiles for therapies with ascite- and dendrite-derived EVs [143]. Since EVs lack the characteristics that cause the mentioned difficulties, several researchers see them as promising candidates for use as therapeutic agents. Future clinical research will almost certainly witness a significant increase in the use of stem cell-derived EVs instead of progenitor cell sources.

## 4. Conclusions

According to this review, MSC-EV therapy has a strong potential for reducing skeletal muscle ageing fragility. Its mode of action, potency, and safety, however, are unknown. As a result, while the promise of MSC-EV application in skeletal muscle ageing frailty is true, more time is needed to optimise culture and processing conditions, as well as understand the regeneration and repair mechanisms. Future trials should be well-designed, i.e., controlled and randomised, with a larger number of patients and a longer follow-up time, to further ensure the safety and efficacy of this unique therapy. Furthermore, further emphasis is needed to improve the dosing regimen, investigate the effect of adjunct therapies on MSC-EV efficacy, and identify molecular biomarkers to signal MSC-EV efficacy.

## Data Availability

Not applicable.
